# Humoral response and neutralising capacity at 6 months post-vaccination against COVID-19 among institutionalised older adults in Argentina

**DOI:** 10.3389/fimmu.2022.992370

**Published:** 2022-09-26

**Authors:** Pamela E. Rodriguez, Andrea P. Silva, Esteban A. Miglietta, Pablo Rall, Carla A. Pascuale, Christian Ballejo, Lucía López Miranda, Antonella S. Ríos, Lila Ramis, Jimena Marro, Verónica Poncet, Bianca Mazzitelli, Melina Salvatori, Ana Ceballos, María M. Gonzalez Lopez Ledesma, Diego S. Ojeda, María F. Aguirre, Yanina Miragaya, Andrea V. Gamarnik, Andrés H. Rossi, Laboratorio SeVa Group, Diana R. Rodriguez García, PAMI Group, Marcela Verónica Alcón

**Affiliations:** ^1^ Fundación Instituto Leloir-CONICET, Buenos Aires, Argentina; ^2^ Departamento Laboratorio de Diagnóstico y Referencia, Instituto Nacional de Epidemiología “Dr. Juan H. Jara”, Mar del Plata, Argentina; ^3^ Instituto Nacional de Servicios Sociales para Jubilados y Pensionados (INSSJP-PAMI), Buenos Aires, Argentina; ^4^ Departamento de Investigación Epidemiológica, Instituto Nacional de Epidemiología “Dr. Juan H. Jara”, Mar del Plata, Argentina; ^5^ Instituto de Investigaciones Biomédicas en Retrovirus y Sida (INBIRS)-CONICET, Facultad de Medicina Universidad de Buenos Aires (UBA), Buenos Aires, Argentina

**Keywords:** SARS-CoV-2, anti-spike IgG, neutralising antibodies, aging adults, AZD1222, BBIBP-CorV, Sputnik-V, Omicron

## Abstract

The COVID-19 pandemic has particularly affected older adults residing in nursing homes, resulting in high rates of hospitalisation and death. Here, we evaluated the longitudinal humoral response and neutralising capacity in plasma samples of volunteers vaccinated with different platforms (Sputnik V, BBIBP-CorV, and AZD1222). A cohort of 851 participants, mean age 83 (60-103 years), from the province of Buenos Aires, Argentina were included. Sequential plasma samples were taken at different time points after vaccination. After completing the vaccination schedule, infection-naïve volunteers who received either Sputnik V or AZD1222 exhibited significantly higher specific anti-Spike IgG titers than those who received BBIBP-CorV. Strong correlation between anti-Spike IgG titers and neutralising activity levels was evidenced at all times studied (rho=0.7 a 0.9). Previous exposure to SARS-CoV-2 and age <80 years were both associated with higher specific antibody levels. No differences in neutralising capacity were observed for the infection-naïve participants in either gender or age group. Similar to anti-Spike IgG titers, neutralising capacity decreased 3 to 9-fold at 6 months after initial vaccination for all platforms. Neutralising capacity against Omicron was between 10-58 fold lower compared to ancestral B.1 for all vaccine platforms at 21 days post dose 2 and 180 days post dose 1. This work provides evidence about the humoral response and neutralising capacity elicited by vaccination of a vulnerable elderly population. This data could be useful for pandemic management in defining public health policies, highlighting the need to apply reinforcements after a complete vaccination schedule.

## Introduction

In the wake of the COVID-19 pandemic, Argentina has incorporated vaccination with a wide variety of vaccine platforms, including the non-replicating adenovirus vaccines AZD1222 (ChAdOx1-S), Ad5-nCoV, and Sputnik V; the mRNA vaccines BNT162b2 and mRNA-1273; and the inactivated SARS-CoV-2 vaccine BBIBP-CorV into its public health policies as the main tool of primary prevention against the disease (1).

In this context, older adults, including those residing in nursing homes, were the second group in order of priority for vaccination, after healthcare workers. These institutions house a particularly vulnerable population, as aging constitutes a relevant and critical risk factor for COVID-19, associated with a higher rate of hospitalisation and death (2, 3). Moreover, this subpopulation presents numerous comorbidities (4) and the semi-enclosed condition of nursing homes tends to facilitate the propagation of SARS-CoV-2.

Argentina initiated in December 2020 its massive vaccination program with three main vaccines: Sputnik V, BBIBP-CorV (Sinopharm) and AZD1222 (Oxford/AstraZeneca). All three platforms studied have shown high efficacy in preventing severe forms of the disease and deaths caused by COVID-19 (5–7). Towards the end of 2020, new viral variants emerged around the world, representing a new challenge against antibody protection generated by infection or vaccination (8). Of these variants of concern (VOC), Omicron is the predominant variant in circulation in Argentina since December 2021 (9).

Given the gap in knowledge regarding the immunological response to vaccines over time among older adults, a prospective multi-centric cohort study was conducted in nursing home residents from the province of Buenos Aires, Argentina. The aim of this research report was to characterise the humoral immune response and neutralising capacity in residents immunised with different vaccine platforms against COVID-19 followed up to 6 months post-vaccination.

## Materials and method

### Target population

This prospective, multi-centric cohort study recruited nursing home residents aged 60 years or over from the province of Buenos Aires, Argentina, between March and May 2021. Nursing homes of Argentina’s National Institute of Social Services for Retirees and Pensioners (PAMI) Local Management Units from the cities of Mar del Plata, La Plata and Lanús (Buenos Aires) which had not started vaccination against COVID-19 at the beginning of the study were considered eligible. Residents who voluntarily agreed to receive two doses of a COVID-19 vaccine and signed the informed consent were included; those with contraindications for venipuncture were excluded.

Enrolled nursing homes were visited by PAMI personnel and residents were vaccinated on-site. Participants received one of three vaccines -Sputnik V, BBIBP-CorV or AZD1222- as part of a homologous prime-boost regimen. The recommended interval between doses outlined by the national program was a minimum of 21 days for Sputnik V, 21-28 days for BBIBP-CorV and 28-84 days for AZD1222 (10, 11). However, as a result of Argentina delaying the second dose until universal coverage with the first dose was achieved, the second dose was applied with variable intervals [median interval (interquartile range) = 62 (55–73), 32 (30–50) and 49 (45–52) for Sputnik V, BBIBP-CorV and AZD1222 respectively].

The following dosage were used: 1x10^11^ viral particles per dose of Sputnik V, 6.5 U of inactivated SARS-CoV-2 antigens per dose of BBIBP-CorV, and 5×10^10^ viral particles per dose of AZD1222 (Manufacturers’ recommendations).

At baseline, demographic data, as well as information on chronic health conditions and previous exposure to SARS-CoV-2 were obtained from 851 participants.

### Samples

Sequential plasma samples were collected at five time points: before vaccination (baseline), 21 days after the first dose, 21 days after the second dose, and at day 120 and 180 after the first dose. Volunteers were divided into two groups according to their self-reported history of COVID-19 and IgG anti-Spike levels at baseline: infection-naïve (without a history of COVID-19 and seronegative at baseline) or convalescent (with a history of COVID-19 or seropositive at baseline).

### Anti-spike IgG levels and SARS-CoV-2 neutralising capacity

We used a non-competitive, semi-quantitative ELISA assay for the detection of specific IgG antibodies against the S protein of the SARS-CoV-2 virus. This assay uses plates coated with a mixture of the trimeric glycosylated Spike protein and its receptor binding domain (RBD) (COVID-AR IgG, CONICET-Leloir-Lemos S.R.L. 2020). The presence or absence of antibodies in the sample was determined by comparing against a cut-off value, according to the specifications of the manufacturer (12). Reactive samples were then titrated using the same assay with serial dilutions in fetal bovine serum (FBS). For the semi-quantitative analysis, the antibody titer was determined as the highest reactive dilution between 1/50 and up to 1/409,600.

Plasma neutralising capacity was assessed on a subset of samples from each vaccine group (Sputnik V= 50, BBIBP-CorV= 49, AZD1222= 50), randomly selected from infection-naïve residents who had at least one seropositive sample. Plasma samples were heat inactivated at 56°C for 30 min. Serial dilutions of the plasma samples were pre-incubated with the VSV-Spike pseudotyped virus (CoV2pp-GFP, ancestral SARS-CoV-2 lineage B.1) (13) for 1 hour at 37°C, and then with cultured VERO cells for 24 hs, according to (14). Neutralisation assays were performed in biological duplicates. The result of this assay was expressed as the dilution that reaches 50% inhibition of infection (IC50) (15).

To study the humoral response against Omicron variant (GISAID accession ID EPI_ISL_10633761) relative to the ancestral B.1 (GISAID accession ID EPI_ISL_499083), neutralising assays were performed using live SARS-CoV-2 virus isolates. A subset of samples from previous neutralising assay who had positive (Sputnik V= 15, BBIBP-CorV= 14, AZD1222 = 15), was selected. Serial dilutions of plasma from 1/4 to 1/8192 were incubated for 1 h at 37°C in the presence of virus in DMEM, 2% FBS. Then, 50 µl of the mixture were added to a Vero cell monolayer for 1 h at 37°C (MOI, 0.01), after which the infectious medium was removed and replaced for DMEM, 2% FBS. After 72 hs, cells were fixed with 4% paraformaldehyde (4°C, 20 min) and stained with crystal violet solution in methanol. The cytopathic effect (CPE) on the cell monolayer was assessed visually. If damage to the monolayer was observed in the well, it was considered as manifestation of CPE. Neutralising titer was defined as the highest plasma dilution without any CPE in two of three replicate wells.

### Quantification and statistical analysis

Descriptive statistics were used to characterise socio-demographic variables and comorbidities of the target population at baseline, according to vaccine. Categorical variables were presented as counts and percentages and their differences evaluated using Chi-squared or Fisher´s exact tests. Missing data were not imputed.

In order to characterise the humoral immune response and neutralising capacity, we considered the following variables: vaccine (Sputnik V, BBIBP-CorV, AZD1222), previous exposure to SARS-CoV-2 (infection-naïve/convalescent), gender (male/female) and age (<80 years/≥80 years). Results were expressed as % of seroconversion upon completion of vaccination scheme, the Geometric Mean (GM) of IgG antibody titer and its 95% confidence interval, pseudotyped neutralising antibody titer (IC50), SARS-CoV-2 neutralising titer (IC80) and its 95% confidence interval. Seroconversion was considered as any increase to detectable levels of antibodies from previous non-reactive samples. Non-parametric (Wilcoxon-Mann-Whitney) tests were used to compare groups. A p < 0.05 was considered statistically significant. For multiple comparisons, Holm’s method (16) was applies to adjust the p-value. We estimated Spearman’s correlation among IC50 pseudotyped virus neutralising titers and IgG anti-Spike titers (0: no relationship, between 0 and ±0.3: weak relationship, between ±0.3 and ±0.7: moderate relationship, between ±0.7 and ±1.0: strong relationship, ± 1.0: perfect relationship). The regression lines between the measures were estimated by Deming regression Statistical analysis and graphical presentations were carried out with R 4.2.0 in the R Studio 1.4.1717 environment (17), using various packages (18–21).

### Ethics

This research was carried out in line with the Guide for Investigation with Human Beings, Resolution 1480/11. Each subject agreed on participating in this research and signed an informed consent prior to any procedure. Participants’ anonymity was maintained throughout the whole study (Law 25.326 of Personal Data Protection). The study protocol was approved by the Research Ethics Committee of the Bernardo Houssay Hospital from Mar del Plata.

## Results

The study evaluated 851 nursing home residents that received one of the following homologous vaccine platforms: Sputnik V (n=522), BBIBP-CorV (n=165) or AZD1222 (n=164); of the initial cohort, 693 participants reached the end of the follow-up (18.6% loss). The immune response was evaluated by measuring IgG anti-Spike titers using a well-established ELISA test (12) and neutralising capacity against a VSV-Spike pseudotyped virus (14), as well as against live ancestral B.1 and Omicron SARS-CoV-2 strains. General information about participants enrolled in this cohort, including gender, age, previous exposure to SARS-CoV-2 and comorbidities by vaccine is listed in **Table 1**. As shown in the table, significant differences were observed in age, gender, previous exposure to SARS-CoV-2 and comorbidities such as heart failure and chronic kidney disease.

**Table 1:** Comparison between patients receiving Sputnik V, BBIBP-CorV and AZD1222 vaccines in terms of demographics, comorbidities, and previous exposure of COVID-19 infection.

Characteristics	N^1^	Sputnik V	BBIBP-CorV	AZD1222	p-value^2^
**Vaccinated**	851	522	165	164	
**Gender n, (%)**	851				<0.001
Female		361 (69%)	132 (80%)	136 (83%)	
Male		161 (31%)	33 (20%)	28 (17%)	
**Age n, (%)**	851				0.015
< 80 years		209 (40%)	48 (29%)	52 (32%)	
≥ 80 years		313 (60%)	117 (71%)	112 (68%)	
**Comorbidities**		**n/n data available, (%)**			
**Diabetes mellitus**	695	68/452 (15%)	9/105 (8.6%)	18/138 (13%)	0.2
**COPD**	697	28/451 (6.2%)	3/107 (2.8%)	5/139 (3.6%)	0.2
**Severe obesity**	697	29/452 (6.4%)	4/107 (3.7%)	7/138 (5.1%)	0.5
**Heart failure**	696	70/451 (16%)	20/107 (19%)	9/138 (6.5%)	0.011
**Hypertension**	696	254/453 (56%)	63/106 (59%)	80/137 (58%)	0.8
**Chronic kidney disease**	697	18/452 (4%)	11/107 (10%)	6/138 (4.3%)	0.025
**Immunodeficiency**	698	10/453 (2.2%)	3/107 (2.8%)	4/138 (2.9%)	0.8
**Previous exposure** **to SARS-CoV-2**	851	251 (48%)	57 (35%)	69 (42%)	0.008

COPD, Chronic obstructive pulmonary disease; Values are represented as number (percent); ^1^Total analysed; ^2^Pearson’s Chi-squared test; Fisher’s exact test.

The cohort presented a mean age of 83 (range, 60-103) years old. The infection-naïve group included 474 participants, while 377 were in the convalescent group. From the latter group, 62% of participants (n=233) did not report infection, albeit displaying specific IgG anti-Spike antibodies. Both groups showed a biased gender (77% and 70% female, in the infection-naïve and convalescent groups, respectively).

**Figure 1:**
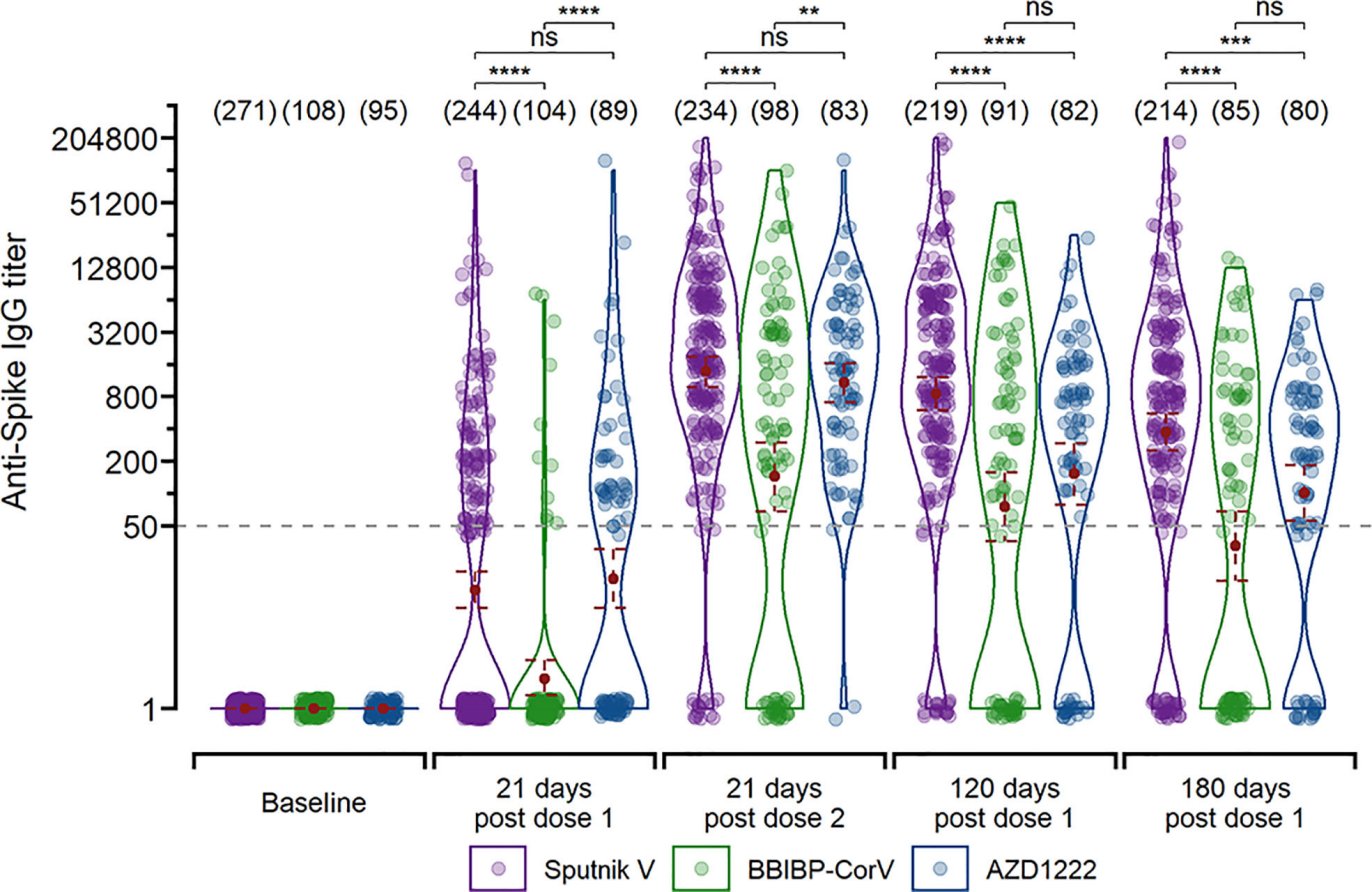
Longitudinal IgG titers after vaccination with different vaccine platforms for infection-naïve participants (n=348). Measurements are shown 21 days after the first dose, 21 days after the second dose, and 120 and 180 days since the first dose for individual that received the two-dose regimen. The GMT with 95% confidence interval are shown. Wilcoxon -Mann-Whitney unpaired U test: ****p ≤ 0.0001; ***p ≤ 0.001; **p ≤ 0.01; “ns”p>0.5.

The immunogenicity after a single vaccine dose in the infection-naïve group widely varied according to the vaccine platform, showing seroconversion rates of 43.6%, 10.6% and 50.0% for Sputnik V, BBIBP-CorV and AZD1222, respectively. The second vaccine dose normalised IgG seroconversion to 92.7%, 68.4% and 97.6%, respectively, with a total 88.1% seroconversion rate. The IgG geometric mean titers (GMT) at 21 days after the second dose were 1374 (CI95%, 983 to 1900) for Sputnik V, 145 (CI95%, 69 to 294) for BBIBP-CorV, and 1087 (CI95%, 697 to 1646) for AZD1222 (**Figure 1**). The GM titers dropped 3.7-fold for Sputnik V, 4.4-fold for BBIBP-CorV, and 10.8-fold for AZD1222, at 6 months after the two-dose vaccination scheme.

On the other hand, the convalescent group showed higher IgG GMT after the first dose than those observed in the naïve group after two vaccine doses for all three platforms. The IgG GMT after the first dose were 12795 (CI95%, 9518 to 16763) for Sputnik V, 2583 (CI95%, 1838 to 3630) for BBIBP-CorV, and 14217 (CI95%, 9948 to 19896) for AZD1222 (**Figure S1**). Unlike the infection-naïve group, these titers did not increase after the second vaccine dose. At 6 months post-vaccination, the IgG GMT of the convalescent group dropped 2.6-fold for Sputnik V, 8.9-fold for BBIBP-CorV, and 3.4-fold for AZD1222.

**Figure 2:**
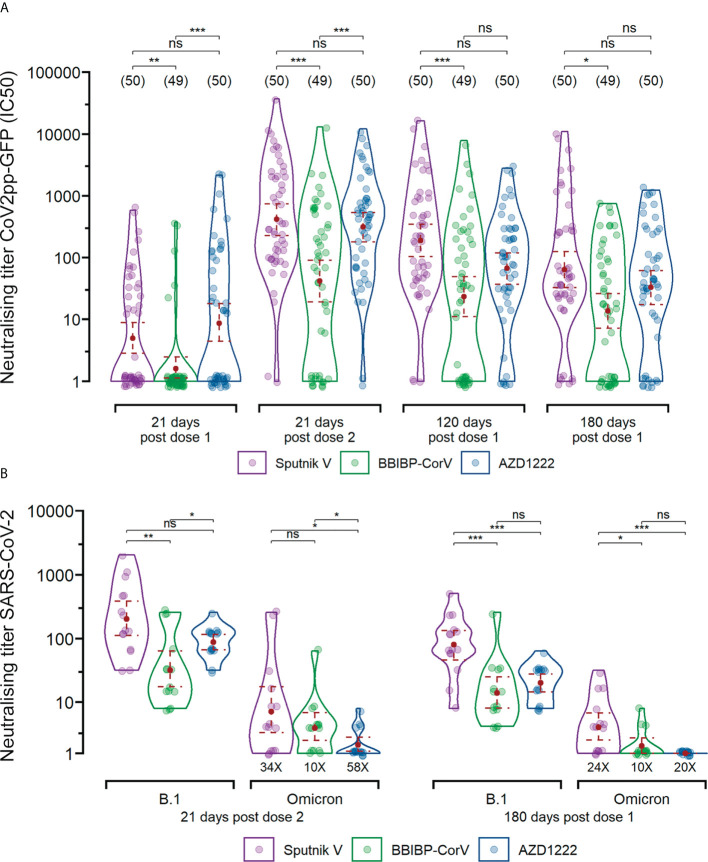
Neutralising capacity after vaccination with different vaccine platforms for infection-naïve participants. **(A)** Titers were measured at 50% inhibition against the pseudotyped B.1 linage virus (CoV2pp-GFP). Measurements are shown 21 days after the first dose, 21 days after the second dose, and 120 and 180 days since the first dose for individual that received the two-dose regimen. **(B)** Using live SARS-CoV-2 virus isolates, titers were defined as the highest plasma dilution without any cytopathic effect on the monolayer. The assay was carried out using B.1 lineage virus and Omicron variant virus. The GMT with 95% confidence interval are shown. Wilcoxon-Mann-Whitney unpaired U test: ***p ≤ 0.001; **p ≤ 0.01; *p ≤ 0.05; “ns” p>0.5.

Analysis of the neutralising capacity against the VSV-Spike pseudotyped virus in the infection-naïve group showed that GM half-maximal neutralising titer (GMT IC50) for Sputnik V was higher than for BBIBP-CorV for all analysed time points, whereas AZD1222 was higher than BBIBP-CorV only up to 21 days after the second vaccine dose (**Figure 2**). Neutralising capacity increased after the second dose, reaching a GMT IC50 of 417 (CI95%, 230 to 745) for Sputnik V, 42 (CI95%, 19 to 91) for BBIBP-CorV and 314 (CI95%, 180 to 537) for AZD1222. Similar to anti-Spike IgG GMTs, neutralising capacity steadily decreased 3 to 9-fold up to 6 months after vaccination for all platforms (**Figure 2A**). The IC50 of pseudotyped virus presented a high and significant correlation with anti-Spike antibodies for all time points analysed (rho= 0.73, 0.85, 0.87, and 0.90 for 21 days post dose 1, 21 days post dose 2, 120 and 180 days post dose 1, respectively) (**Figure S2**).

Neutralising antibody titer against Omicron resulted lower (10-58 fold) compared to B.1 for all vaccine platforms at 21 days post dose 2 and 180 days post dose 1 (vaccinated with 2 doses). For both time points GM neutralising titer (SARS-CoV-2) for Sputnik V (GM= 6.0, CI95% 2.2 to 17.5 and GM= 3.3, CI95% 1.7 to 6.4, respectively) was higher than for BBIBP-CorV (GM= 3.3, CI95% 1.7 to 6.4 and GM= 1.4, CI95% 0.9 to 2.1, respectively) and AZD1222 (GM= 1.5, CI95% 1.0 to 2.3 and GM= 1, respectively) (**Figure 2B**).

**Figure 3:**
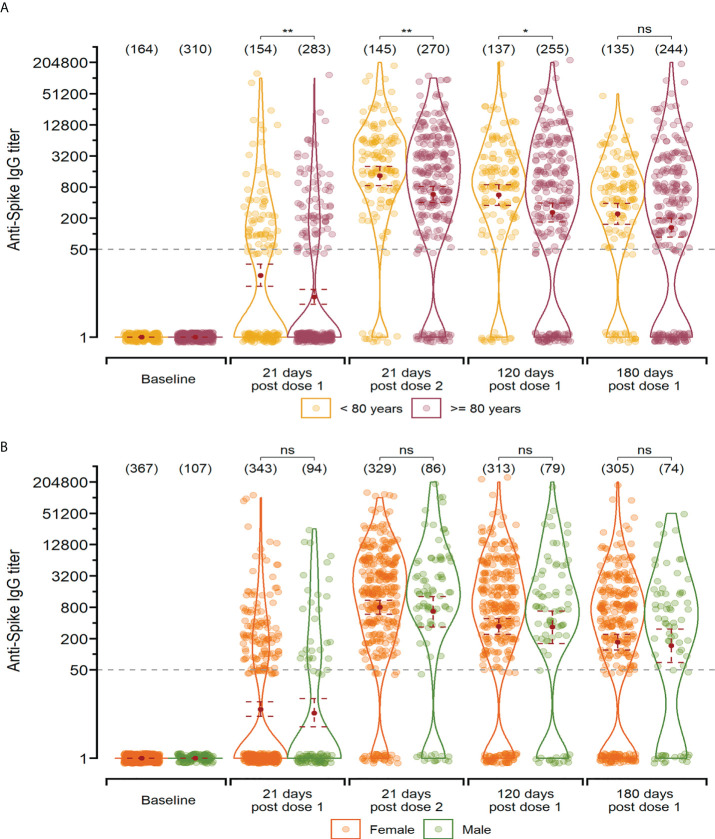
Anti-Spike IgG titers after vaccination in infection-naïve participants segregated by age-group **(A)** and gender **(B)**. Measurements are shown at baseline, 21 days after the first dose, 21 days after the second dose, and 120 and 180 days since the first dose for individual that received the two-dose regimen. The geometric mean with 95% confidence interval are shown. Wilcoxon-Mann-Whitney unpaired U test: **p ≤ 0.01; *p ≤ 0.05; “ns” p>0.5.

When analysing all vaccine platforms together, infection-naïve residents aged 60-79 (<80 years) showed significantly higher anti-Spike IgG titers after vaccination, compared to residents over 80 (**Figure 3A**). In contrast, the convalescent group presented no significant differences between the age groups (**Figure S3A**). When participants were segregated by gender, no significant differences were observed in the naïve group (**Figure 3B**), while convalescent residents presented a marginal difference (*p* = 0.0229) in anti-Spike IgG titers biased towards men at 120 days after vaccination (**Figure S3B**). Analysing the infection-naïve group, a very weak inverse correlation was observed between age and anti-Spike IgG antibody titer only in those participants who received BBIBP-CorV (rho= -0.23 p= <0.05). No correlation was observed between age and antibody neutralising capacity against the pseudotyped virus (**Figure S4 A, B**).

## Discussion

To date, there is still a significant gap in knowledge regarding the immune response elicited by the Sputnik V, BBIBP-CorV and AZD1222 vaccines in the older adult population, particularly in Argentina (22, 23). Here, we assessed the anti-Spike IgG and viral neutralising humoral response, both of which are relevant readouts of vaccination efficacy (24–26). We show that all three vaccine platforms were efficient in developing a humoral response in the older population studied. We report that both specific anti-Spike IgG titers and SARS-CoV-2 neutralising capacity increased after the second dose. Moreover, we show that both previous exposure to SARS-CoV-2 and age (<80 years) are factors associated with higher antibody titers.

Of note, we determined that over 50% of the convalescent population studied had not been aware of their exposure to the virus (did not declare history of COVID-19), but were seropositive at baseline. This result could prompt revising of prevention strategies against the spread of infection in semi-enclosed environments housing risk groups, such as nursing homes.

As mentioned previously, nursing home residents were among the highest groups in priority for vaccination. Thus, in order to accelerate the rate of coverage, the different vaccine platforms were applied according to their availability. This context meant that the groups analysed here, were not defined *a priori*. Moreover, comorbidities were not considered as an exclusion criterion for participating in the study. As a consequence, we found differences in demographic characteristics (age, sex) and prevalence of comorbidities among vaccine groups such as heart failure and chronic kidney disease. The lower rate of participants with heart failure in the AZD1222 group could be attributable to some contraindications for this platform for subjects with heart disease. However, the position adopted by the European Society of Cardiology was that vaccination was indicated for all patients with heart failure, as they state that “corrective measures should not be allowed to delay vaccination” (27). Although kidney transplant patients have a poor immune response to vaccination, those with chronic kidney disease, even at stages 4-5, have been reported to present antibody levels comparable to healthy controls (28, 29).

The rates of seroconversion for the infection-naïve group in our population of older adults were similar to those reported for the general population for both Sputnik V (5) and AZD1222 (6, 30, 31) but lower than the rate previously reported for people over 60 years vaccinated with BBIBP-CorV (32, 33). This data is valuable because aged individuals are at higher risk of experiencing more severe forms of COVID-19, as well as hospitalisations and death. To illustrate, in 2020, people aged 60 or over represented 85.5% of the total deaths related to COVID-19 in Argentina (Argentinian National Ministry of Health, 2022).

After completing the vaccination schedule, infection-naïve residents who received either Sputnik V or AZD1222 exhibited significantly higher humoral responses than those who received BBIBP-CorV. Other studies comparing the latter with Sputnik V (34) and others found similar results (35, 36). Conversely, in the convalescent group, a booster effect was observed after the first dose for all three platforms, suggesting the existence of immunological memory, as reported for other vaccines (37–40). Interestingly, in this same convalescent group, the second dose of either of the three vaccines did not further boost antibody levels. Rossi and collaborators (15) found similar results in healthcare workers immunised with Sputnik V, as did others in adults immunised with AZD1222 and different vaccines platforms (30, 41).

In line with previous results reported for Sputnik V (14) and others (42), we observed a progressive waning in specific antibody titers up to 6 months after immunisation. Particularly, Oviedo-Rouco and collaborators (43) showed that antibody levels in older adults with a homologous scheme for BBIBP-CorV decrease significantly from 21 up to 220 days post vaccination. These and other similar observations prompted many countries, including Argentina, to implement booster doses to improve population-wide immunity towards 2021 and 2022 (Resolution 1426/21 National Ministry of Health).

Similar to anti-Spike IgG titers, neutralising capacity against the VSV-Spike pseudotyped virus also significantly increased after the second dose of the vaccines in infection-naïve residents for all vaccine platforms and showed a progressive waning over time. Regarding viral neutralising capacities, titers were lower for BBIBP-CorV than for Sputnik V and, to a lesser extent, for AZD1222. Several reports analysing the same vaccine platforms studied here (15, 44) and others, such as mRNA-1273/Moderna (45), Ad5-nCoV/Covidicea (46) and BNT162b2/Pfizer–BioNTech (44, 47) observed increased neutralising activity after the second doses on ancestral and several VOCs. As published in other reports with vaccines analysed here (31, 48), we observed a positive linear correlation between neutralising capacity and anti-Spike IgG. Thus, a growing amount of evidence suggests that relative anti-Spike IgG titers can be used as a predictor of neutralising activity. Nonetheless, Hernandez-Bello et al. described a tendency towards a negative correlation between these variables (46).

Similarly to several previous studies, Omicron was capable to escape the humoral response induced by the vaccine platforms analysed here in resident with complete scheme of vaccination and 6 month later (49, 50). This evidence indicates that the original 2-dose vaccine schemes are insufficient to protect against Omicron, one of the most widely spread variants worldwide. Oviedo Rouco et al. demonstrated that heterologous booster doses markedly increased neutralising activity against this VOC in older adults who had received two doses of BBIBP-CorV.

Regarding the influence of advanced age on the immune response after vaccination, we observed higher specific antibody titers in infection-naïve subjects aged 60-79 years after the first dose of the vaccines compared to subjects over 80. A similar difference in efficacy has been reported for the BNT162b2/Pfizer-BioNTech vaccine (51), although neutralising antibodies were still detectable after the second dose, independently of age (26). In contrast, neutralising antibody titer against VSV-Spike antibody did not significantly vary between age groups nor did it correlate with age, as previously reported for the CanSino vaccine (46). Similarly, neutralising titers for both B.1 and Omicron observed in our population of older adults was comparable to those reported by Pascuale et al. for volunteers with a mean age of 41 years who received the same three vaccine platforms (52).

This work provides useful information regarding the humoral response and neutralising capacity elicited by Sputnik V, BBIBP-CorV and AZD1222 vaccines in older adults. The significant difference in neutralising efficacy between the ancestral B.1 and Omicron strains shows that this pandemic is a dynamic phenomenon and, as such, requires response strategies to also be dynamic and continually updated. We consider that this knowledge is valuable to inform decision making in public health, particularly about the importance of implementing booster strategies for improving protection, especially against VOCs such as Omicron.

## Laboratorio SeVa Group

Diana R. Rodriguez García, Magalí G. Bialer, María José de Leone, Natalí B: Rasetto, Shirley D. Wenker, Luciana Bianchimano, Maria Soledad Treffinger Cienfuegos, C. Esteban Hernando, Daniel A. Careno, Corina Garcia.

## PAMI Group

Marcela Verónica Alcón, Diego Sossa Centurión, Candela Raffo Velázquez, Noelia Inés Aztorga, Romina Solazzi, Fernando Miguel Bacigaluppe, María Soledad Fernández, Hernán Salaya, Eduardo Perez.

## Data availability statement

The raw data supporting the conclusions of this article will be made available by the authors, without undue reservation.

## Ethics statement

The studies involving human participants were reviewed and approved by Research Ethics Committee of the Bernardo Houssay Hospital from Mar del Plata. The patients/participants provided their written informed consent to participate in this study.

## Author contributions

Conceptualisation and experimental design: AHR, YM, APS, and AVG; collection of plasma samples and clinical data: PR, and PAMI Group; IgG anti-spike titer determination: PER, APS, EAM, CAP, LLM, ASR, LR, VP, Lab SeVa Group, DSO, and MFA; determination of neutralising titers using CoV2pp GFP pseudotyped virus: PER, EAM, and MMGLL; determination of neutralising titers using live virus: BM, MS, and AC; data curation and analysis: PER, EAM, PR, CAP, CB, JM, MFA, and AHR; manuscript drafting: PER, APS, EAM, PR, CAP, CB, JM, MFA, and AHR, with corrections from AVG; funding acquisition: AVG. All authors contributed to the article and approved the submitted version.

## Funding

This work has received funding from *Fondo para la Convergencia Estructural del Mercosur* (FOCEM) to AVG, *Fondo Nacional para la Investigación Científica y Tecnológica de Argentina* PICT 2019-02869 to AVG, NIH U19AI168631-01 to AVG. National Institute of Epidemiology “Dr. Juan H. Jara”, received funding from the Argentine government. Founds for this work were also provided by *Fundación Williams* to AVG.

## Acknowledgments

The authors would like to thank all the voluntaries who participated in this study for their selfless collaboration, as well as to the personnel from PAMI and the nursing homes who were instrumental in acquiring and transporting the samples.

## Conflict of interest

The authors declare that the research was conducted in the absence of any commercial or financial relationships that could be construed as a potential conflict of interest.

## Publisher’s note

All claims expressed in this article are solely those of the authors and do not necessarily represent those of their affiliated organizations, or those of the publisher, the editors and the reviewers. Any product that may be evaluated in this article, or claim that may be made by its manufacturer, is not guaranteed or endorsed by the publisher.

## Supplementary material

Figure S1 Longitudinal IgG titers after vaccination with different vaccine platforms for convalescent participants. Measurements are shown at baseline, 21 days after the first dose, 21 days after the second dose, and 120 and 180 days since the first dose for individual that received the two-dose regimen. The GMT with 95% confidence interval are shown. Wilcoxon-Mann-Whitney unpaired U test: ****p ≤ 0.0001; **p ≤ 0.01; “ns”p > 0.5.

Figure S2 Correlation between neutralising titer CoV2pp-GFP (IC50) and IgG anti-Spike titer at different time post vaccination. Spearman’s correlation: 0, no relationship; between 0 and ±0.3, weak relationship; between ±0.3 and ±0.7, moderate relationship; between ±0.7 and ±1.0, strong relationship; ±1.0: perfect relationship.

Figure S3 Anti-Spike IgG titers after vaccination in convalescent participants segregated by age-group (A) and gender (B). Measurements are shown at baseline, 21 days after the first dose, 21 days after the second dose, and 120 and 180 days since the first dose for individual that received the two-dose regimen. The geometric mean with 95% confidence interval is shown. Wilcoxon-Mann-Whitney unpaired U test test: **p≤0.01; *p≤0.05; “ns” p>0.5.

Figure S4 Correlation of antibodies titer by age in residents vaccinated with different platforms (Sputnik V, BBIBP- CorV and AZD1222). A. IgG anti-Spike titers vs. age in infection-naïve group (n = 474). B. Antibody neutralisation levels (IC50) vs. age (n = 149). Spearman’s correlation was performed. A p < 0.05 was considered statistically significant.
