# Supplementary figures and images for: Humoral response and neutralising capacity at 6 months post-vaccination against COVID-19 among institutionalised older adults in Argentina
Source: Front Immunol. 2022 Sep 26;13:992370. doi: 10.3389/fimmu.2022.992370 (PMC9549602; doi:10.3389/fimmu.2022.992370)

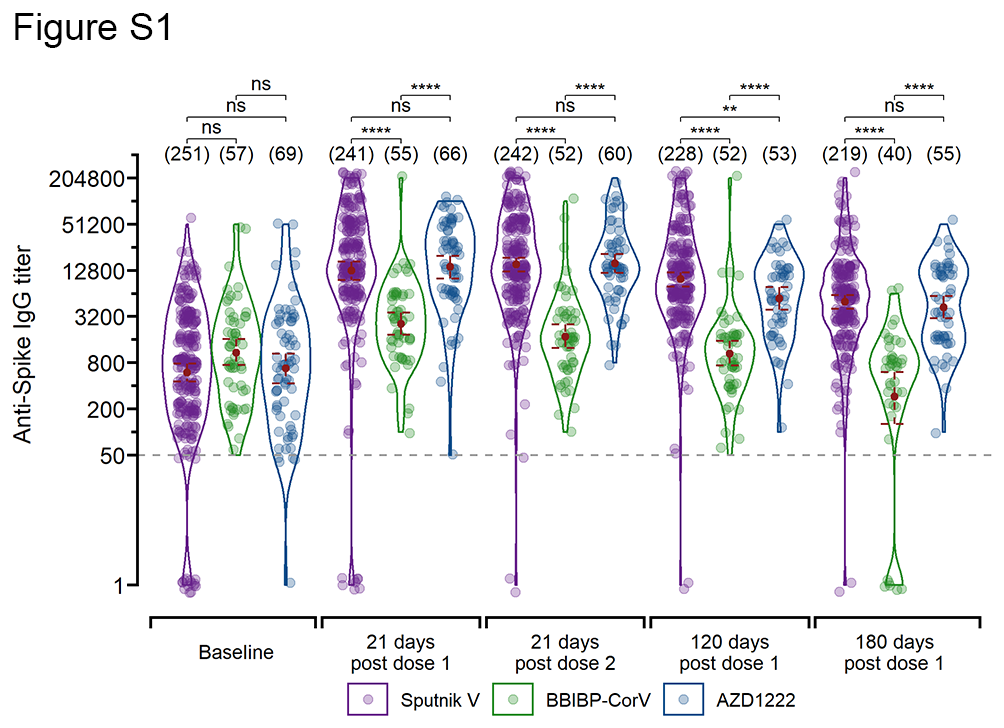

Supplement: Figure S1 — Longitudinal IgG titers after vaccination with different vaccine platforms for convalescent participants. Measurements are shown at baseline, 21 days after the first dose, 21 days after the second dose, and 120 and 180 days since the first dose for individual that received the two-dose regimen. The GMT with 95% confidence interval are shown. Wilcoxon-Mann-Whitney unpaired U test: ****p ≤ 0.0001; **p ≤ 0.01; “ns”p > 0.5. [file Image_1.tif]

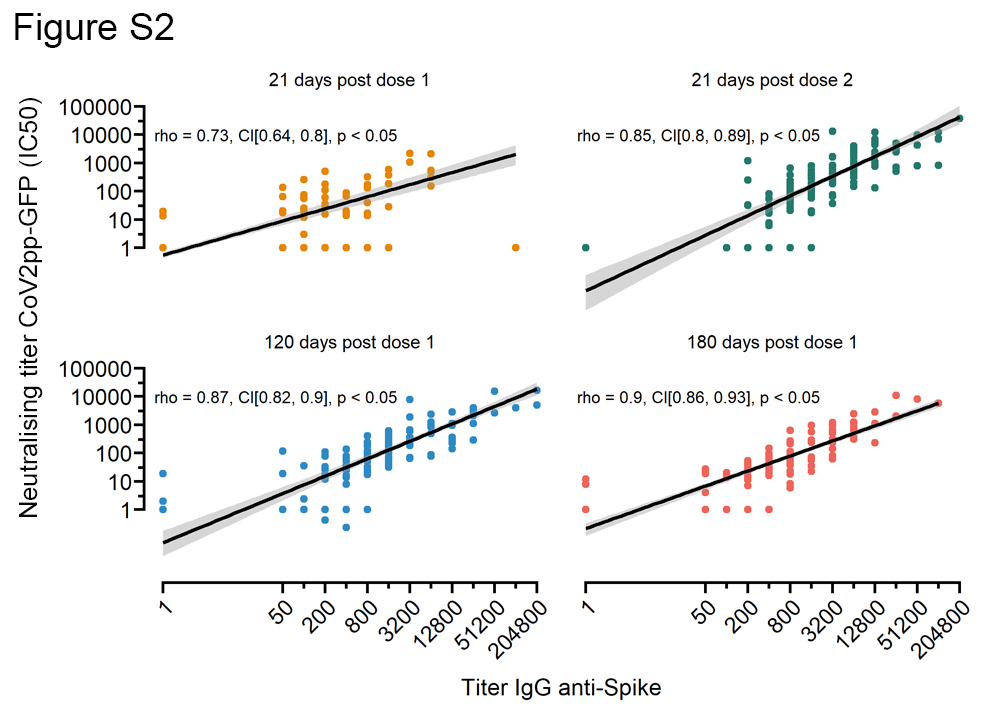

Supplement: Figure S2 — Correlation between neutralising titer CoV2pp-GFP (IC50) and IgG anti-Spike titer at different time post vaccination. Spearman’s correlation: 0, no relationship; between 0 and ±0.3, weak relationship; between ±0.3 and ±0.7, moderate relationship; between ±0.7 and ±1.0, strong relationship; ±1.0: perfect relationship. [file Image_2.tif]

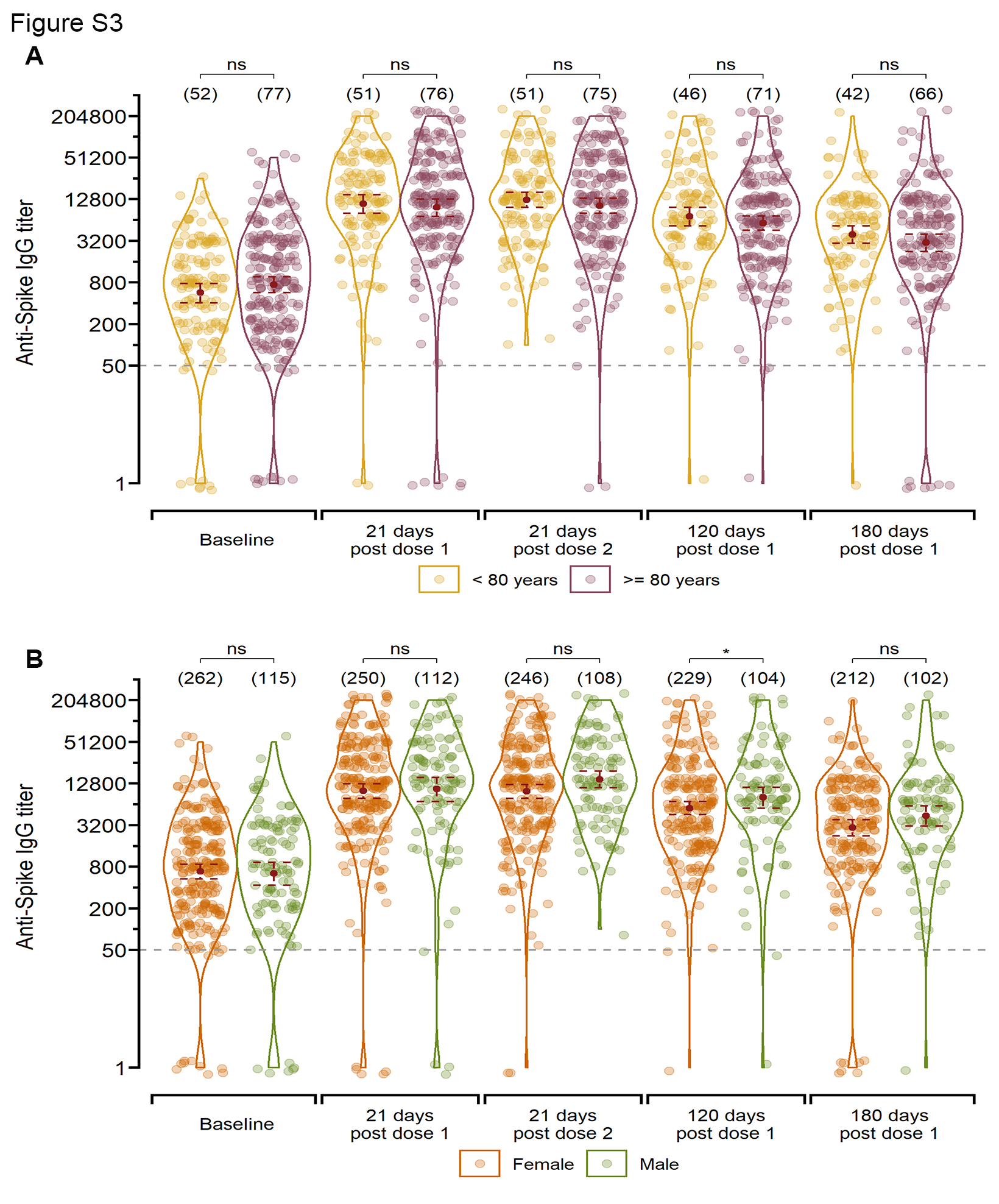

Supplement: Figure S3 — Anti-Spike IgG titers after vaccination in convalescent participants segregated by age-group (A) and gender (B). Measurements are shown at baseline, 21 days after the first dose, 21 days after the second dose, and 120 and 180 days since the first dose for individual that received the two-dose regimen. The geometric mean with 95% confidence interval is shown. Wilcoxon-Mann-Whitney unpaired U test test: **p≤0.01; *p≤0.05; “ns” p>0.5. [file Image_3.tif]

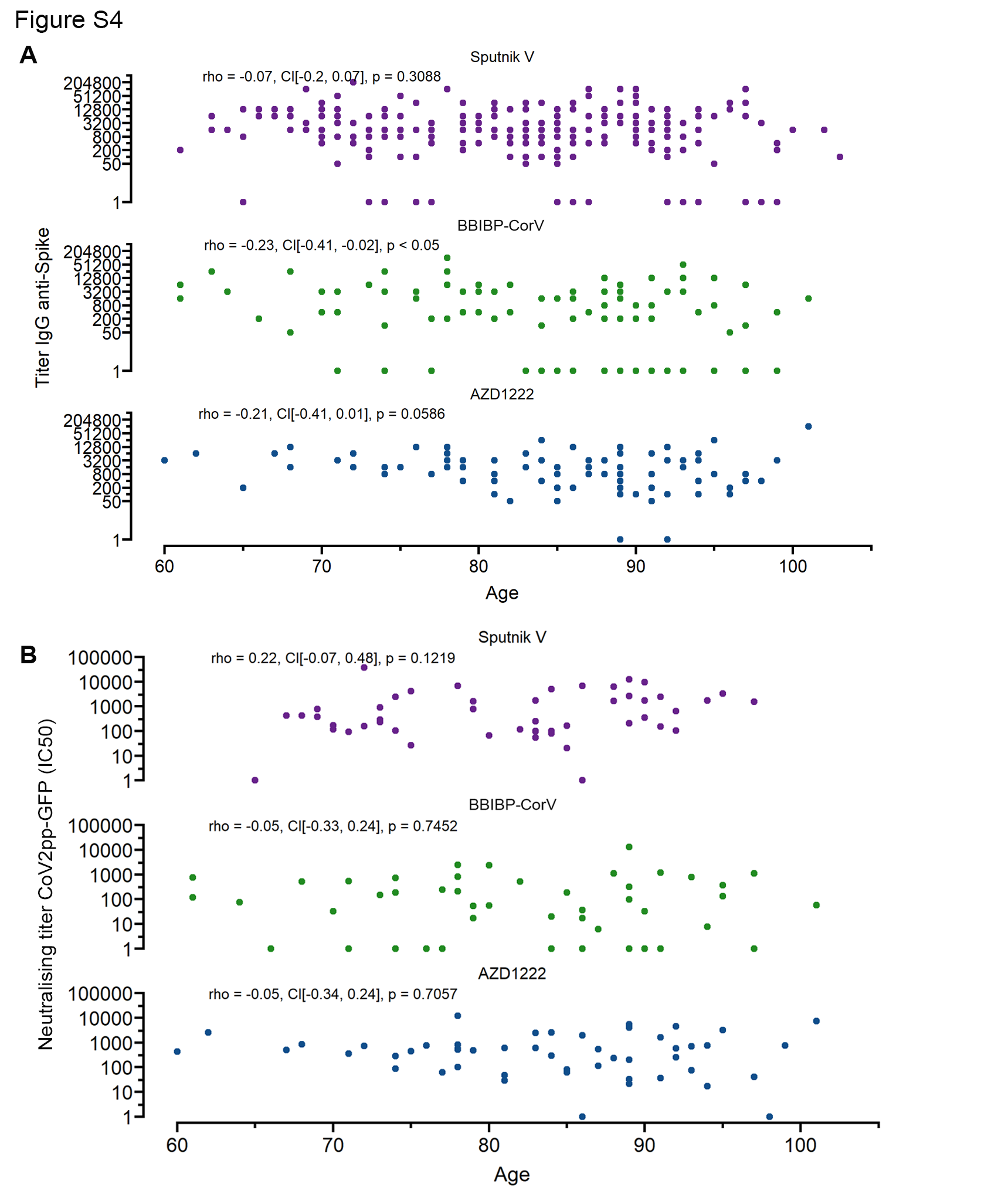

Supplement: Figure S4 — Correlation of antibodies titer by age in residents vaccinated with different platforms (Sputnik V, BBIBP- CorV and AZD1222). A. IgG anti-Spike titers vs. age in infection-naïve group (n = 474). B. Antibody neutralisation levels (IC50) vs. age (n = 149). Spearman’s correlation was performed. A p < 0.05 was considered statistically significant. [file Image_4.tif]
